# Barriers to timely treatment-seeking in patients with acute myocardial infarction in Malaysia: a qualitative study

**DOI:** 10.1186/s12912-016-0155-5

**Published:** 2016-05-28

**Authors:** Li S. Chai, Zabidah Putit, Sidiah Siop

**Affiliations:** Department of Nursing, Faculty of Medicine and Health Sciences, Universiti Malaysia Sarawak (UNIMAS), Jalan Datuk Mohd Musa, 94300 Kota Samarahan, Sarawak Malaysia

**Keywords:** Barrier, Thoughts, Beliefs, Acute Myocardial Infarction, Delay, Decision time

## Abstract

**Background:**

Persisting delay in seeking treatment among Acute Myocardial Infarction (AMI) patients was reported in Malaysia despite intensified efforts in educating the public on symptoms of AMI and the importance of seeking prompt treatment. Studies outside Malaysia have shown that patients’ personal thoughts during symptom onset could contribute to the delay. The purpose of this study is to explore the barriers of AMI patients prior to the decision of seeking treatment in Malaysia.

**Methods:**

A qualitative descriptive research approach was chosen. Individual in-depth interviews were conducted among 18 patients with AMI. Data were analysed using thematic analysis. Recordings were transcribed and coded, codes were subsequently organized into categories. The stages of coding and identifying categories were repeated before themes were identified.

**Results:**

Three meaningful themes with nine sub-themes that may have influenced the delayed decision to seek treatment were identified. Some themes identified were culturally bound.

**Conclusions:**

The findings of this study give insights on barriers prior to the decision of seeking treatment when patients were experiencing AMI. Findings indicates that interventions targeted at increasing knowledge about AMI symptoms and correct actions using an informative approach at the current practice may not be adequate to reduce patient delay. The findings of this study could provide basis for the development of interventions that are culturally relevant to the Malaysians setting to promote behavioural change in the population and reduce pre-hospital delay.

## Background

Coronary Heart Disease caused 2.3 million deaths globally in 2012 [1]. In Malaysia, it remains the leading cause of death from 2000 to 2012, and accounts for 20.1 % of all deaths in 2012 [2]. Acute Myocardial Infarction (AMI) is the most deadly presentation of Coronary Heart Disease. In-hospital and 30-day mortality following AMI is high at 7 and 13 % respectively in 2010 [3].

In the management of AMI, delay in seeking treatment after symptom onset could have great consequences on prognosis. Early reperfusion with thrombolytic therapy has been shown to reduce mortality by up to 50 % when it is given within two hours from symptoms onset [4–7]. In Malaysia, thrombolytic therapy constitutes the main reperfusion strategy (74 %) in the treatment of AMI due to limitation of resources for angioplasty in the local hospitals [3]. Efforts have been intensified in Malaysia to reduce the delay to treatment including educating public on symptoms and importance of early treatment for AMI [8]. Despite these efforts, the in-hospital mortality rate for AMI was reported to remain consistent between 8 and 10 % from 2006 to 2010 and it was higher compared to other global registries within the same period [3]. Delay to treatment after symptoms onset has been found as the main cause of high mortality for AMI [8]. A constant mean of this delay was reported between 3.7 and 4.4 h from 2007 to 2012 [9]. In addition, a proportion of AMI patients did not receive any form of primary revascularization therapy because of late presentation leading to missed opportunity for the appropriate therapy [3].

Symptom onset in this study is defined as the episode of symptoms that has become constant. The symptoms can range from typical chest pain, pain or discomfort in other areas of upper body, to atypical presentations like dizziness, dyspnoea, diaphoresis, weakness, syncope, nausea, and vomiting [10]. Patient delay is the delay from symptom onset to first medical contact [11]. Patient delay represents a combination of two delay intervals: decision time (onset of symptom to decision to seek treatment), and transportation time (decision to seek treatment to hospital arrival). Decision time has been described as the longest interval of delay [12–14]. Improvement could be achieved if factors influencing the delay in this interval are examined. Numerous studies have shown that knowledge about AMI symptoms and the awareness to seek early treatment is not the only determinant influencing the decision time. Studies have described the patients’ personal factors like feelings, emotions, behaviour, and reactions towards AMI symptoms contributed to the delay in decision time [13, 15–19]. Ethnic and cultural differences have also been considered to affect the delay time [14, 20–23].

The context of this study is in Sarawak, which is Malaysia’s largest state. The only government hospital that provides cardiac care service in the state is Sarawak Heart Centre, with eight percent of the total number of heart disease patients in Malaysia being treated in the hospital from 2007 to 2010 [3]. Unpublished data showed that almost half of the AMI patients admitted to Sarawak Heart Centre had a longer delay of more than three hours (Statistic Unit: Time to treatment in AMI, patients admitted from 2010 to 2014, unpublished). In our literature search using databases that include Medline, Cochrane, ScienceDirect, and Cumulative Index to Nursing and Allied Health Literature (CINAHL), few studies described causes of in-hospital delay to treatment in Malaysia, including admission procedures [24], late referral to cardiologist [25, 26], and inappropriate triage [25]. However, there was no research found in relation to factors influencing the decision time. As there might be cultural, belief, and values differences in the ways AMI symptoms are interpreted in a Malaysian setting, the purpose of this study is to explore the barriers prior to the decision of seeking treatment of AMI patients in Malaysia. We hope that findings from this study will provide insights on patient delay in prehospital phase and assist in improving the current implementations that target at reducing patient delay.

## Methods

A qualitative descriptive research approach [27] was chosen because very little is known about the barriers of AMI patients in Sarawak prior to seeking treatment. Individual in-depth interviews were conducted by the first author to gain detailed understanding of the thoughts and meaning constructed by patients when experiencing AMI.

### Setting

Participants were obtained from the Coronary Care Unit, Sarawak Heart Centre. Purposive sampling was used in this study and the inclusion criteria were patients who were diagnosed with AMI, who were stable hemodynamically, and chest pain-free for at least 48 h. Upon obtaining consent, the patients were interviewed two to five days after the admission. Interviews were conducted in the patients’ cubicle. The setting of Coronary Care Unit enabled a private room for the interviews because each cubicle is only occupied by one patient.

### Participants

Characteristics of the participants are shown in Table 1. During the data collection period, nineteen male and four female patients were approached. One male and two female patients declined to be interviewed. Eighteen patients participated in this study, and amongst the participants, only one was a female patient. The low presentation of female patients could be due to predominantly male population with AMI as the demographic report in Malaysia showed that male patients accounted for 85 % of AMI in 2010 [3]. However, this should not pose a concern because this study is not designed to compare the findings between male and female patients. The mean age of the participants was young at 47 years. AMI patients in Malaysia were typically younger when compared with other Western registries and the demographic report in 2010 indicated that 31 % of AMI patients were aged less than 50 years at presentation [3]. The mean decision time was 204 min (3.4 h), and the longest was 1140 min (19 h). Benchmarking technique described in other studies [18, 28–30] was used in this study to verify the time during symptom onset or when decision to seek treatment was made. It was also found to be useful when participants could not recall the time. In these cases, they were asked to relate the time to routine daily events, such as meals, bedtime, or favourite television programme.

**Table 1:** Characteristics of the participants

Characteristics	
Participants	18
Patient delay (minutes), mean, *range*	
Onset to decision to seek care	204, *40-1140*
Decision to seek care to hospital arrival	24, *5-50*
Age, mean, range	47, *31-70*
Sex	
Male	17
Female	1
History of angina pectoris	4
Previous AMI	0
Religion	
Christian	4
Muslim	6
Buddhist	2
Taoism	4
None	2
Ethnic	
Iban	4
Chinese	7
Malay	6
Indian	1

### Questions guideline

Interviews conducted in this study were primarily guided by questions guideline. It is a written list of open-ended questions that need to be covered during the interview. It was designed based on research objective, related literatures, clinical working experience, and improved as interview progressed. The exact order and wording of the questions were different from each patient, and interview conducted always followed new issues that arose. The first main question usually was ‘can you tell me about the first signs you experienced when you had your heart attack’. The subsequent questions were designed to understand the aspects that had delayed their decision to seek treatment. These questions were about the patient’s beliefs and thoughts before any actions that may prolong the decision time. Under these questions, there were probing questions to ensure relevant data could be generated, for example ‘can you tell me more specifically about that?’

### Data collection

Data collection was conducted from July 2012 to February 2013 by the first author. Interviews usually started with greetings and personal conversation. When participants showed their readiness, interview and recording began. Interviews ranged from 21 to 44 minutes. There was no time limit imposed but the interviewer tried to complete the interview within one hour as not to interrupt the ward routine and patients’ rest time. The use of questions guideline helped in the decision on how to utilize the time available and helped the interviewer to elicit data needed from the participants. At the same time, interviews were flexible enough to allow new probing questions as the need arise. The interviewer kept in mind to be respectful, non-judgmental, and non-threatening during interview sessions. Interviews were carried out depending on participants’ preferred language, and were conducted in Malay language for 11 participants and Mandarin for seven participants. The interviewer is familiar and fluent with these two commonly spoken languages in Malaysia. Phone calls were made to 11 participants, who had been discharged from the Coronary Care Unit for verification of interviewer’s interpretations.

Data analysis began as soon as first interview was conducted and continued in parallel with data collection. After analysing transcribed data from the sixteenth participant, no new codes and categories were found. The first author continued to interview two more participants until no new codes and categories emerged from the seventeenth and eighteenth participants’ transcripts. It was decided then that data has reached saturation after 18 interviews because further coding is no longer feasible in the last three participants’ transcripts [31].

### Data analysis

This study used thematic analysis to analyse the data [32]. Participants’ experience, views, feelings, and emotions that had been recorded were transcribed by the first author using participants’ spoken language directly, where no translation to English was made to preserve its nuances [33]. Some words in Mandarin and Malay languages could not be easily conveyed in English and their meaning would lose in translation. The transcripts were read several times by the first author to obtain a sense of whole. Transcripts were then read word by word. The words, phrases, sentences or paragraphs that appear to capture or describe AMI patients’ experience were highlighted and coded. Codes were organized into categories, based on their relationships.

The first author listened through the recordings again and transcripts were referred. The stages of coding and indexing categories were repeated. Some codes flourished and some codes decayed. The codes were re-examined and re-categorized. Exemplars for each code and category were identified from the data. Translation into English was done by the first author for these exemplars. The second and third authors checked the Malay translations by referring to the transcripts to make sure that the translations were accurate and capture the meanings of original language. The Mandarin translations were checked by a physician, who is familiar with Mandarin and English. All authors met regularly to discuss the emerging ideas until reaching an agreement about the categories. Relationships between categories were further identified among the authors with focus to find the consistent and recurrent patterns between categories.

### Methodological rigor

In qualitative research the concepts credibility, dependability, transferability and confirmability have been used to describe various aspects of trustworthiness [34, 35]. To achieve credibility, participants were encouraged to be honest and given the opportunities to decline the research participation. Throughout the process of data collection, the first author kept a memo to write down notes and observations. Memo was referred to during analysis and it helped to conceptualize the data in hand. Member checking was carried out during and after the interviews [36]. During the interviews, the first author would summarize what the participants said and allowed them to immediately correct errors of interpretations. Phone calls were made to 11 participants to verify interviewer’s interpretations during the analysis process. All interviews were recorded, fully transcribed and the first author kept the research aim in mind during the process of coding the data to ensure no relevant data is excluded and no irrelevant data is included. The recordings were listened through again, and transcripts were found accurate in the step reinterpreting categories in data analysis. Agreement was sought from two experts in research methods with the way the data were labelled and sorted. To approach dependability, questions guideline was used to serve as a guideline of questions to be asked or issues to be probed during interview. To strengthen transferability, excerpts were shown to point out the original data in transcripts, where categories were formulated. To achieve confirmability, similar findings from other studies were referred to in the discussion of the findings.

## Results

The findings consist of three themes that described the participants’ thoughts in the decision phase: ‘hesitation to seek treatment’, ‘denying the symptoms’, and ‘believing in fate’. Table 2 presents the themes and sub-themes.

**Table 2:** Themes and sub-themes in the decision phase of patients with AMI

Sub-themes	Themes
Uncertain about their symptoms	Hesitation to seek treatment
Uncertainties to ask for another help	
Persuading themselves that symptoms were not life threatening	Denying their symptoms
Belief of being capable to control the symptoms	
Belief of being capable to bear the pain	
Holding on to their responsibilities	
Dealing with expectations	
Accepting death	Believing in fate
It was not time yet	

### Hesitation to seek treatment

#### Uncertain about their symptoms

The participants described the feeling of uncertainty about their symptoms when experiencing AMI. They said they never encountered such symptoms before and did not know what was happening to them. They were taking their time as they were uncertain whether their symptoms need medical treatment or not:

‘*I never had this pain before. I kept asking myself what is this? I didn’t go to the hospital, because I didn’t know what I was experiencing’* (Lily^1^).

The participants debated with themselves in relation to how serious their condition was. They were not sure how much pain they should experience before seeking medical care. These feelings of uncertainties took a great deal of time:

‘*The pain started at six in the morning … I never had this pain before, never… I didn’t know why my chest was painful. I was thinking was it serious? I didn’t feel like going to the hospital yet because I could still bear with it. It was until nine at night that the pain was getting stronger*’ (Zainudin).

The pain origin was also described at the gastric region, neck, jaws, shoulder and few participants said they only experienced weakness. The inconsistencies between what they felt and their understanding of a heart attack contributed to the hesitation to ask for help:

‘*I was having weakness only that day. I had no pain. I felt weakness here* (pointing at his left hand), *and here* (pointing at his left shoulder), *and here* (pointing at his right shoulder). *How would I know it was a heart attack? If it was a heart attack, I would feel the pain at my chest right*?’ (Muthu).

#### Uncertainties to ask for another help

Few participants said they sought help in general practitioner’s clinic for their symptoms and was told that they were experiencing gastritis. They were reassured that medicine prescribed for them from the clinic would reduce their pain. However, they found their symptoms continued and worsened after they took the medicine. They spent much time to convince themselves that the medicine prescribed could not relieve their pain. They also described their struggles to bear with the pain:

‘*I went home after seeing Dr X. I had no appetite for dinner, but I took the medicine. I wanted to sleep earlier at nine, but I couldn’t sleep. The pricking pain was there since morning and I bore with it. Maybe the medicine hasn’t started to work yet. At four in the next morning, the pain was more intense and I couldn’t bear with it anymore*’ (Chong).

### Denying their symptoms

#### Persuading themselves that symptoms were not life threatening

The participants described that at the beginning, they tried to reason out that their symptoms were related to feeling of too tired, indigestion, gastritis, dizzy spells, and air ‘trapped’ in their chest. When the pain became intense, they took some time persuading themselves the symptoms were not serious enough to seek treatment:

‘*I told myself maybe I was having dizzy spells because I fell down when I tried to get up. So I didn’t seek any help yet. I was just having giddiness’* (Darrel).

Some participants also expressed that they tried to reassure themselves that the symptoms would subside and would be similar to their previous experience of angina, which only lasted for a while.

#### Belief of being capable to control the symptoms

Participants described that they believed they could control the symptoms. They tried remedies and thought the remedies could reduce their pain:

‘*The first thing I thought when I had the pain was, I want my throat to feel warm so I drank warm water believing that the pain would go away’* (Baidul).

The participants also said that they took time to see whether the remedies were effective or not. The remedies included drinking water, taking bath, massaging, taking a rest, sleeping, and taking medication such as gastric pill and Chinese’s herbs. Some of them tried few remedies:

‘*When I had the pain after dinner about seven in the evening, I tried to stand up… but I sweated. So I sat on a chair. I called my wife and she massaged my back and my chest. I was still having the pain so I tried to walk around. Then I applied ‘Vick’ ointment on my chest. I looked at the clock and it was already midnight, the ‘Vick’ ointment didn’t reduce my pain*’ (Amin).

They eventually sought help when they realized that they could not control the pain and the remedies were not working.

#### Belief of being capable to bear the pain

Participants confessed that they were struggling to tolerate with their pain. They tried to hide the pain by not telling people around them during the attack. They continued activities like doing housework, driving, playing Mahjung games, and activities at work. They described that they tried their best to endure the pain until they could not bear with it anymore before the decision to seek help and that took some time:

‘*The pain was getting stronger every minute. I bore with it until I almost fainted*’ (Zainudin).

Participants also explained that the pain was bearable due to their high pain threshold:

‘*I could still bear with it that time. I’m not scared of pain. I’m the one who can bear the highest pain threshold.*’ (Bong).

#### Holding on to their responsibilities

Participants described they chose to bear the pain they were experiencing because they wanted to complete the tasks given to them at their work place. They said they were still able to work despite the symptoms they had. They expressed that they needed to work and to be paid as they were the breadwinners of their family. Participants also confessed that they were putting their responsibilities first. They said that they were accountable for their work and expected themselves to carry out the responsibilities:

‘*That fateful day at around three in the afternoon, I felt a bit of chest pain and difficulty in breathing, then I felt I was going to faint, but I stayed calm, then I continued to work until five. I told my boss, it’s almost five, I shall finish my work first*’ (Kenneth).

There were expressions of worries if they could not carry out their duties:

‘*I didn’t go to the hospital. I don’t know. I thought about my family, because we make noodles to earn money. There was a lot of work that I needed to do and they needed me*’ (Lily).

#### Dealing with expectations

The participants expressed that they never thought of any serious illness when they were experiencing the symptoms because they were still young and healthy. They said they could not relate their symptoms to a heart attack despite the pain at their chest. The participants also described that while someone told them their symptoms could be a heart attack, they responded as not believing the advice:

‘*It never came across my mind that it was a heart attack. I am young you know. Even though after my mother told me it was, I still believed in some way that it was not*’ (Hoon).

They convinced themselves that by practising a healthy lifestyle, they were not at risk of having heart diseases:

‘*I believe I wasn’t experiencing a heart attack that time. I heard about it before, but I didn’t think about it. I didn’t think the symptoms were related to it. I jog every day and I can jog for one and a half hour without rest.//I always eat fish and vegetables*’ (Philip).

### Believing in fate

#### Accepting death

Some participants said that they took time to bear with their symptoms because of their belief in fate. This was especially expressed by those of Islamic faith. They realized the pain they had could be life threatening when it became worse. However, they said they were not scared and believed the pain was beyond their control. They described the belief about the power of God that controls and decides their death:

‘*The pain went to my back and my neck. I thought it might be time, but I leave it to Allah. Allah knows when I would die, not me. When it is time, we wouldn’t wake up. I wasn’t scared. I accepted my fate and whatever Allah would do to me. I’m His umrah, His people. He’s the leader*’ (Rahman).

Participants believed they could not change the situation if it was their fate to die. They said they accepted death and did not seek treatment:

‘*If Allah doesn’t want to give any more time for me, no matter how others help me, how much efforts they use, I would also die… I leave it to Allah… I search for my inner peace. I leave it to Allah, let it be the way Allah wanted to… If Allah still wants to see me tomorrow, then I would survive’* (Asnul).

Participants also expressed that they gave their last words to their family members while accepting death:

‘*I told my wife, I would have to go* (die) *first. You go on with your life. Our sons have grown up. They can take care of you. My sons were crying when they heard me saying this. I told my sons how I love their mother and I asked them to take care of her like the way I did’* (Baidul).

#### It was not time yet

The belief in fate was also expressed in different ways. Few participants who practised Taoism mentioned that they were not worried that their pain would cause death. They described that they believed it was not time for them to die yet despite the intense symptoms. They also said that the pain they had would disappear and there was no urgency to seek treatment:

‘*If I were to die, I have already died a long time ago, but I survived many events. The time has not come yet. It’s true. We don’t need to worry about being dead or alive. If you are destined to die, you won’t miss it. If you are not, you wouldn’t have any problem*’ (Chong).

## Discussion

In this study, the aim was to explore the barriers of AMI patients prior to the decision of seeking treatment in Malaysia. It is hoped the results of this study would provide understandings on the persisting patient delay despite the efforts to increase the awareness of AMI. There are numerous studies examining the barriers outside Malaysia, but the issue has never been studied in the Malaysian setting before. Given that the data were collected until saturation was reached, this study highlights the barriers that may be applicable to many people with similar culture who are experiencing an AMI.

One of the main findings was the uncertainties about the AMI symptoms. The participants in this study described how they hesitated and were unsure of how much pain they should have before they seek help. The hesitation because of uncertainties was also described in two studies in the western countries [18, 37] and such lack of clarity of the symptoms was found central to the delay in seeking treatment. Participants in this study had difficulty in recognising the symptoms as a health threat that requires immediate treatment. For those who consulted general practitioners and were reassured that their symptoms would subside, a period of time was needed by them to verify the need for treatment. In a study that investigated the role of uncertainty in diagnostic information, even a minor reduction in certainty would decrease the urgency of response for high severity threats [38]. The findings in this study suggest that in order to reduce patient delay, certainty of having AMI symptoms is essential during symptom onset. Improvement is necessary in the efforts to educate both the patients and the general practitioners. These efforts require careful planning of effective messages about the typical and atypical AMI symptoms that could occur to help them to recognize and interpret the symptoms correctly.

Under the theme denying their symptoms, findings show that participants denied the pain for few reasons. These were frequently expressed together with the self-persuasion that the symptoms were not serious and would subside. Experiencing sudden AMI symptoms was unexpected for the participants in this study and they never thought of any serious illness because they believed they were still young, healthy, and practising healthy lifestyle. The lack of perceived susceptibility to heart disease has also been described in AMI patients in other countries [39–41] and such somatic under awareness was associated with longer delays [42, 43]. When the participants had such belief, the symptoms were not perceived as a threat. This simple message is lacking in the current health education in Malaysia. It should be emphasized to raise the awareness of AMI risk factors and hopefully would increase the perception of risk for AMI in the population.

Many of the participants in this study were the breadwinners of their family and thus had enormous responsibilities. They had to work to survive and they denied sickness when experiencing the symptoms. This findings were similarly described in two studies in Hong Kong and Korea [14, 23], which found the unique self-sacrificing behaviour in Asian population who tried to maintain their social responsibilities when afflicted by AMI symptoms. Participants in this study tried to fulfil their duties and phrases like ‘shall finish my work first’, ‘thought about family’, or ‘worried if not done’ showed similar self-sacrificing behaviour. Interventions to reduce delay should consider this finding and effort should be made to modify the behaviour which may have prolonged the patients’ decision time to seek treatment.

The findings in this study suggest that perceived control during the episode of AMI may be one of the significant barriers to reduce delay in treatment-seeking. Participants expressed their beliefs of being capable to control symptoms, which led to many self-care remedies. This also can be explained in a way that when participants realized the pain was beyond their control, they then decided to seek treatment. Perceived control is a personal belief that individual has controllability on behalf of one’s self and ability to control threats [44]. Higher perceived control reduced anxiety and is associated with a better psychological and physical outcome in patients with cancer [45], persistent mental illness [46] and after myocardial infarction [47]. However, it is different in relation to seeking medical care after the onset of AMI symptoms. Few studies found that a strong personal sense of control predicted longer delay time [48–50]. Hence, behavioural change of individual’s perceived control may be beneficial in the efforts to reduce the delay.

The findings also suggest that participants in this study demonstrated pain resistance behaviour. Participants described how they clenched their teeth and endured the pain until it became unbearable. They explained that they viewed themselves as having the ability to bear the highest degree of pain. Studies in Brazil and Egypt have shown that the pain resistance behaviour was a significant predictor of prolonged prehospital delay in AMI patients [19, 51]. This behaviours could be influenced by the complexities of cultures [52]. The demographics of Malaysia are represented by the multi ethnic groups consisting of Malays and Bumiputera (67.4 %), Chinese (24.6 %), Indians (7.3 %), and others (0.7 %) [53]. Each ethnic in the country has its own distinct culture. There was limited research on cultural attitudes of pain among the multi-ethnic group in Malaysia. Nevertheless, in a study conducted in Singapore, a country that shares similar cultural heritage with Malaysia [54], it was found that Malay ethnicity had lower pain severity compared with Chinese, and Indian had greater pain severity when compared with both Malay and Chinese ethnicity [55]. The description of cultural influence on the pain resistance behaviour might be limited in this study. However, the thought that they could endure the pain appears to be an important barrier to reduce delay in seeking treatment.

The findings in this study suggest that cultural factor may have influenced the treatment-seeking decisions among the different ethnics of participants in this study. In the findings, one of the reasons for chest pain mentioned by the participants was that there was air trapped in their chest. This description is similar to the findings of one study which was carried out among Malaysian Chinese in a suburban population [56]. Chinese participants in the study believed that joint pain was caused by excessive ‘wind’ accumulated in the joint and abdominal colic was caused by excessive ‘wind’ in the abdomen. This concept of air trapping or excessive wind is embedded in the Malaysian Chinese who practised Taoism [56]. Taoism is mostly practised by Chinese and constitutes 1.3 % of the religion in Malaysia [53]. Taoist believe the *yin* and *yang* forces, and disease is a result of an imbalance or blockage in these forces [57]. The influence of the culture could also be seen when participants tried Chinese’s herbs to reduce their pain. Many patients who use traditional Chinese medicine believe that modern medicine is not holistic because it disregards the interaction between the individual and the environment [57]. The findings of this study suggest that such belief may affect the individual response to AMI symptoms and delay the decision to seek appropriate treatment.

To our knowledge, accepting death when experiencing severe pain in AMI has not been described before. In the theme believing in fate, some participants described they were not afraid of death and did not take the initiatives to seek treatment because they believed death is unavoidable and is controlled by Allah in their Islam religion. The findings contrast with prior studies in other countries, in which the thought about intense symptoms would bring death eventually triggered the feeling of fear in their patients and prompted the early decision to seek treatment [58–60]. In a qualitative study conducted in Turkey, AMI patients who described the development of fear of death were effective in their decision-making process and sought immediate treatment [61]. In the context of this study, however, participants’ decision to seek treatment seems to be delayed by their belief in fate. Islam is the most widely professed religion in Malaysia with 61.3 % of its population is Muslim [53]. The concept of fate is fundamental in Islam. Muslim believes that Allah creates human beings, determines their life span and death [62]. Death may become a less stressful event for a Muslim when he truly believes in Islam teaching [63]. Such beliefs, however could be one of the barriers to seek treatment and may be difficult to change.

Fate was also expressed by participants who practised Taoism but its description was different from the participants of Islam religion. They believed they would survive the event despite the severe symptoms because it was not yet their fate to die and hence no urgency to seek treatment. Taoists believe that part of their life is predestined and unchangeable [64]. Death is natural and to fear death is seen as meaningless. What happens after death is not important to Taoists, whose ambition is immortality, the prolongation of physical life [65]. Such beliefs of fate may also be constructed by participants’ personal experience as they mentioned they have survived many events, and this reinforced their reluctance to seek treatment. The findings of this study imply that the awareness of AMI and getting prompt treatment is not the only contributing factor influencing the decision time. The culturally bound beliefs may have influenced their decision of seeking treatment in hospital and could be an important obstacle at the efforts to reduce pre-hospital delay.

### Limitations

Limitations of the present study include the potential recall bias. Patient recalled their thoughts few days before during the interviews. Besides, this study did not recruit AMI patients who did not seek medical care, patients who were not stable to be interviewed during data collection, and patients who died outside the hospital. These patients who could not be recruited might also experience the barriers to seek care.

## Conclusions

Persisting pre-hospital delay in AMI patients over the past few years was reported in Malaysia. The findings of this study may provide explanation for the persisting delay as the themes identified give insights on patients’ barriers prior to the decision of seeking treatment when experiencing AMI. The barriers include the uncertainties of their symptoms, denial, self-sacrificing behaviour, perceived control over their symptoms, pain resistance behaviour, and culturally bound beliefs. These responses towards AMI symptoms and culturally bound beliefs may have influenced the patients’ decision to seek treatment. This suggests that current informative approach based on simple messages about knowledge of symptoms, and to get early treatment in the event of these symptoms may not be sufficient to reduce patient delay and need to be reviewed. The findings of this study could provide the basis for the development of interventions that are culturally relevant to the Malaysians setting to promote behavioural change in the population and reduce the pre-hospital delay.

## Abbreviation

AMI, acute myocardial infarction

## Footnotes

1 All participants’ names were pseudonyms in this study to ensure confidentiality.
